# Optimal Timing of Cardioverter-Defibrillator Implantation in Patients with Left Ventricular Dysfunction after Acute Myocardial Infarction

**DOI:** 10.31083/j.rcm2304124

**Published:** 2022-04-02

**Authors:** Andreea Maria Ursaru, Irina Iuliana Costache, Antoniu Octavian Petris, Mihai Stefan Cristian Haba, Ovidiu Mitu, Adrian Crisan, Nicolae Dan Tesloianu

**Affiliations:** ^1^Department of Cardiology, Emergency Clinical Hospital “Sf. Spiridon”, 700111 Iași, Romania; ^2^1st Medical Department, “Grigore. T. Popa” University of Medicine and Pharmacy, 700111 Iași, Romania

**Keywords:** early ICD implantation, sudden cardiac death, myocardial infarction arrhythmias, primary prevention, low ejection fraction heart failure, ICD therapies, ICD shock

## Abstract

**Background:**

Prevention of sudden cardiac death (SCD) early after acute myocardial infarction (AMI) is still a challenge, without clear recommendations in spite of the high incidence of life-threatening ventricular arrhythmias, as implantable cardiac defibrillator (ICD) placement is not indicated in the first 40 days after an AMI; this timing is aleatory and it is owed to fact that the two pivotal studies for evaluation of ICDs in primary prevention, MADIT and MADIT II, excluded the patients within three, respectively four weeks after AMI.

**Methods:**

We conducted a retrospective, single-center study that included 77 patients with AMI. All patients were monitored by continuous ECG in the first week after the event. Transthoracic echocardiography was performed at discharge and 40 days after the event. Patients with ejection fraction of 35% or less as assessed by 2D echocardiography 40 days after the MI, which received an ICD for the primary prevention of SCD, were included in the study. The subjects were followed for a median of 38 months, by means of device interrogation and echocardiography.

**Results:**

We divided our patients into two groups: in the first group, with left ventricular ejection fraction (LVEF) under 30% after MI, all patients remained in the reduced ejection fraction heart failure category, with an increase from an initial mean of 18.93 ± 4.99% to a mean of 22.18 ± 4.53% after a period of 40 days; we obtained a positive and statistically significant correlation (*p *< 0.001 and r – 0.547), and all patients presented indication of ICD implant 40 day after MI. In the second group with LVEF between 30% and 35% after MI, the mean LVEF increased from an initial mean of 31.73 ± 1.33% to a mean of 32.33 ± 1.49% after a period of 40 days. A statistically significant correlation (*p* – 0.02 and r – 0.78) was obtained, although 3 patients presented a LVEF over 35% at 40 days post-MI. Most of the ICD therapies (14.54%) appeared in patients with LVEF <30% and these patients also presented a higher percentage of NSVT at initial ECG monitoring (54% vs. 50%) and NSVT at ICD interrogation (80% vs. 66.7%); statistical significance was not reached – *p *> 0.05. The majority of the ICD therapies (11.9% from 13.4%) appeared in patients with NSVT at initial ECG monitoring; also, these presented an increased number of NSVT at ICD interrogation (77.6% vs. 6%) when compared to patients without VT detection at the initial ECG monitoring. Still, statistical significance was not reached – *p *> 0.15.

**Conclusions:**

The patients could benefit from ICD implant earlier than stated in the actual guidelines, since there are insufficient data in the literature for the waiting time of 40 days. Correlated with the increased risk of SCD in the first months post myocardial infarction, the present study proves the benefit of early ICD implantation considering that all our patients with a low ejection fraction immediately after infarction remained in the same category and the great majority (96.1%) required the implantation of an ICD after 40 days. Thus, we could avoid exposing our patients at risk of SCD for an unnecessary prolonged period, and choose early ICD implantation.

## 1. Introduction

Acute coronary syndrome (ACS) represents a major healthcare and economic burden all around the globe, accounting for around 1.8 million deaths (20% of deaths) in Europe and 655,000 deaths in the USA (25% of deaths) yearly [1, 2]. Sudden cardiac death (SCD) is closely related to ACS, as post-mortem studies revealed that 2/3 of the patients who suffer of out-of-hospital SCD present coronary disease. SCD is defined as the sudden, unexpected death secondary to onset of life-threatening loss of cardiac function (sudden cardiac arrest — SCA) [3, 4]. The most common pathophysiological mechanism is ventricular arrhythmia (VA): over 50% of the patients die due to sustained ventricular tachycardia (VT) or ventricular fibrillation (VF) [5]. In conjunction with ACS, SCD can manifest as the initial coronary event in 15% of the patients. Retrospective analysis showed that there is no difference in the incidence of SCD according to the type of ACS: ST-Elevation Myocardial Infarction (STEMI) vs. non-ST-Elevation Myocardial Infarction (non-STEMI), as well as with symptomatic and silent myocardial infarction (MI). As a long-term event, 50% of SCDs are usually encountered in the first year after ACS and 25% in the first three months [6].

Defibrillation therapy is the only tool that has proved to be highly effective in terminating life-threatening VAs, therefore ICDs represent the main solution for the prevention of SCD [7, 8]. Regarding the ICD placement after acute MI, the European Society of Cardiology (ESC) guidelines indicate implantation in patients with reduced ejection fraction heart failure, with a class I, level of evidence A recommendation, unless they have had a MI in the prior 40 days. There is grey zone starting 48 hours after the ACS until the 40th day, period in which a wearable cardioverter-defibrillator (WCD) could be taken into consideration — a IIb level of evidence B recommendation [9]; such therapies appear as an additional expense and expose the patient to multiple risks, including inappropriate therapy, non-recognition of VAs with the consecutive non-delivery of the necessary shocks besides the lack of pacing capabilities or the discomfort of the patient. Even more, WCD are not available in all countries, because these are not reimbursed of some health care system, even in Europe. Considered a high-cost therapy, implanting an ICD, is actually less expensive than associating other bridge therapy devices, such as WCD, that add a supplementary significant initial cost.

The European and American guidelines ground their recommendations against ICD in the first 40 days after MI on the results of two trials, DINAMIT (Defibrillator in Acute Myocardial Infarction trial) [10] and IRIS (The Immediate Risk Stratification Improves Survival) [11] which investigated the role of the defibrillator in the immediate post-acute myocardial infarction (AMI) setting. Although ICDs were associated with a lower risk of SCD in these randomized trials, this was offset by the association with a high risk of non-SCD events. Other trials that influenced the timing of ICD implantation are two pivotal studies for evaluation of ICDs in primary prevention, Multicenter Automatic Defibrillator Implantation Trial (MADIT) [12] and Multicenter Autonomic Defibrillator Implantation Trial II (MADIT II) [13]. The VALsartan In Acute myocardial iNfarcTion (VALIANT) trial [14] enrolled patients with a LVEF ≤40% after MI and demonstrated the risk of SCD in the immediate post-AMI period is the highest in the first 30 days.

Through our study we aim to point out the importance of considering early ICD implantation post-AMI. There is no straight path to follow within the first 40 days after the myocardial infarction, but we should try to avoid exposing the patients with reduced ejection fraction to supplementary risks. Uncovering the hidden findings and after a close, careful and critical analysis of the major trials investigating the need of early ICD, corroborating results from trials as VALIANT and other smaller studies which bring to frontline the importance of arrhythmic SCD in the first month after MI and considering factors that predict life-threatening VAs, our study comes to strengthen the need for early ICD implantation in all patients with LVEF <30% immediately post MI.

## 2. Materials and Methods

### 2.1 Study Design and Patients 

This retrospective study was conducted at a single center in a consecutive series of patients, between January 2017 and April 2021. During this period, 258 patients with ischemic cardiomyopathy were implanted with an ICD for the primary prevention of SCD in our center. Of these, 77 consecutive patients presented to our institution with acute MI and were monitored through continuous ECG recording during the first week after the event. VT occurring within the first 48 hours after AMI was not considered. Transthoracic echocardiography was performed at discharge, and 40 days after the event. Patients with ejection fraction of 35% or less as assessed by 2D echocardiography 40 days after the MI, which received an ICD for the primary prevention of SCD, were included in the study. Median follow-up was 38 months (range, 7 months to 57 months). Patients of either sex who were more than 18 years of age (there was no upper age limit) with clinical heart failure and LVEF equal to or below 35% despite optimal medical therapy after more than 40 days after the AMI were included. New York Heart Association (NYHA) functional class II or III represented inclusion criteria for ICD recipients and NYHA class IV for CRT-D recipients. Exclusion criteria were defined as follows: patients on the urgent waiting list for a heart transplant, human immunodeficiency virus (HIV) positive patients with an expected survival of less than 3 years due to HIV, lack of informed consent, age under 18 years and severe depression or other major psychiatric illness. 7 patients were excluded because of the sustained VT with need of electrical cardioversion, and immediate ICD implant for secondary prevention of SCD.

AMI was defined as present in patients with typical lasting chest pain and increase of cardiac enzymes above the normal range associated with onset of ST-T changes compatible with myocardial ischemia (ST segment elevation or depression, T-wave inversion) or abnormal Q waves. The patients received immediate or selective coronary angiography (CAG) and primary PCI using standard techniques associated with pharmacological treatment or pharmacological treatment only.

Non-sustained VT was categorized as at least 4 consecutive ventricular beats with a rate >150 beat-per-minute (bpm). All ventricular ectopic beats lasting at least 4 beats were reviewed and confirmed by cardiologists. LVEF was calculated using 2D echocardiography by biplane Simpson’s method, obtained by the same observer, in order to avoid the inter-observer variability. The echocardiograms were performed using a GE VividTM V7 ultrasound device (General Electric, Boston, CA, USA).

The study was conducted according to the ethical guidelines of the Declaration of Helsinki, revised in 2013, and it was approved by the Ethics Committee of “Sf. Spiridon” Hospital.

### 2.2 ICD Therapy

Single and dual-chamber ICDs, as well as biventricular devices were implanted. The defibrillation leads were single-coil leads. In order to treat only rapid, sustained VT or VF, the devices were uniformly programmed according to the MADIT-RIT delayed therapy arm (170–199 bmp with 60 s delay; 200–249 bmp with 12 s delay; ≥250 bpm with 2.5 s delay) and the ADVANCE III trial, with longer delay—30 of 40 instead of the conventional 18 of 24, with two or three therapy zones. VT was primarily treated with anti-tachycardia pacing (ATP) and possibly consecutive ICD shocks. VF was primarily treated with ICD shock with ATP during charging. Over time, changes in programming routines have occurred, in order to avoid inappropriate shocks. A “monitor only” VT detection was set at 150 bpm [15, 16]. Appropriate therapy was defined as shock or ATP for real VT or VF following analysis of the intracardiac electrograms.

Because of the potential of pacing to worsen congestive heart failure, the minimal pacing rate was set to 40 beats per minute, without rate-responsive pacing excepting CRT devices [17, 18, 19].

### 2.3 Statistical Analysis 

We used Kolmogorov–Smirnov test for the assessment of the normal distribution of continuous variables in the study population. Normally distributed parameters are presented as mean ± standard deviation and mean ± min/max values; to compare the mean values (in the case of continuous variables) we used the Student’s *t*-test and one-way ANOVA. Categorical variables were presented as frequencies and percentages. The assessment of the correlation between two variables was performed using the correlation coefficients (r) Pearson and Spearman.

Data analysis was performed using IBM SPSS Statistics for Windows (IBM Corp. Released 2021. IBM SPSS statistics for Windows, Version 28.0. Armonk, NY: IBM Corp, USA) and Microsoft Excel (Microsoft Office 2016 for Windows, released by Microsoft, WA, USA) for organizing data before statistical processing. All tests were two-tailed and a *p*-value < 0.05 was considered statistically significant.

### 2.4 Follow-up of The Patients

Follow-up was performed at 40 days after the acute MI, then 1 month, 3 months and then every six months after the ICD implant. The visits consisted of clinical and paraclinical examinations, including transthoracic echocardiography and interrogation of the devices. Clinical surveillance involved monitoring of the patients and anticipated visits in case of occurrence of symptoms, worsening of the clinical status or internal electrical shocks.

## 3. Results

### 3.1 Characteristics of The Patients 

Baseline characteristics, essential clinical and paraclinical data of the patients with acute MI are presented in Table 1. Median age of the patients was 64 years (35–83 years), with a predominance of male sex (69%).

**Table 1.** **Characteristics of patients eligible for ICD implant after the 40 days evaluation (LVEF ≤35%)**.

Characteristic		At admission for MI	At the time of ICD implant
Median NT-proBNP level — pg/mL		2287 (620–3432)	1565 (360–2967)
Median left ventricular ejection fraction, %		25.3	27.3
Median estimated GFR — mL/min/1.73		54 (11–96)	59 (15–98)
Systolic BP (mmHg)		140 (92–178)	122 (111–139)
Diastolic BP (mmHg)		81 (53–102)	72 (63–81)
BMI (kg/$m^{2}$)		28.2 (21.9–35.1)	27.5 (21.8–33.9)
Medication, n (%)			
	Amiodarone	6 (9)	26 (38.8)
	ACE I/ ARB	35 (52.2)	55 (82)
	Beta–blocker	23 (34.3)	64 (95.5)
	Loop–diuretics	21 (31.3)	65 (97)
	Mineralocorticoid–receptor antagonist	19 (28.3)	63 (94)
	ARNI	2 (3)	11 (16.4)
	Dapagliflozin	2 (3)	7 (10.4)
Aspirin		21 (31.4)	67 (100)
DAPT		2 (3)	51 (76.1)
Coexisting conditions, n (%)			
	Hypertension	46 (68.6)	46 (68.6)
	Permanent atrial fibrillation	15 (22.4)	17 (25.4)
	Smoker, n (%)	17 (25.4)	12 (17.9)
	Diabetes mellitus, n (%)	12 (17.9)	12 (17.9)
	Uncontrolled Dyslipidemia, n (%)	53 (79.1)	23 (34.3)
CRT-D patients			12 (17.9)
Bundle branch block			
	Left		14
	≥150 ms		8
	130–150 ms		4
	<130 ms		2
	Right		4
	Indeterminate		2

ACE-I, Angiotensin-converting enzyme; ARB, Angiotensin-receptor blocker; ARNI, Angiotensin receptor-neprilysin inhibitor; BP, blood pressure; BMI, body mass index; CRT-D, Cardiac Resynchronization Therapy Defibrillator; DAPT, Dual antiplatelet therapy; GFR, glomerular filtration rate; Ms, milliseconds; MI, myocardial infarction; NT-pro BNP, N-terminal pro-brain natriuretic peptide.

The patients medication at the index event and at the time of the ICD implant are also mentioned in Table 1. The heart failure, antithrombotic therapy as well as and lipid lowering medications was optimized, along with lifestyle changes recommendations. The majority of subjects received target doses of heart failure medication in accordance with the available guidelines.

From a total of 77 consecutive patients that presented to our institution with acute MI and reduced LVEF (35% or less as assessed by 2D echocardiography), 70 (90.9%) were evaluated at 40 days after the MI, in order to receive an ICD for the primary prevention of SCD. Of these, 67 patients (87%) remained in the category of reduced ejection fraction at 40 days, and as a consequence received an ICD; 7 patients (9.1%) presented VAs with hemodynamic instability and required ICD implantation earlier, for the secondary prevention of SCD. Of the initial 77 patients, 74 patients (96.1%) required the placement of an ICD. All descriptive information can be analyzed in Table 2.

**Table 2.** **Statistical description of patients with reduced ejection fraction (<35%) after myocardial infarction**.

Initial number of patients, n	77
Patients with sustained VT necessitating external defibrillation — ICD implantation for secondary prevention before the 40th days, n (%)	7 (9.1)
Patients evaluated 40 days after the index event, n (%)	70 (90.9)
Patients eligible for ICD implant after the 40 days evaluation (LVEF ≤35%), n (%)	67 (87)
Total number of patients that received ICD, n (%)	74 (96.1)

ICD, implantable cardiac defibrillator; LVEF, left ventricular ejection fraction; VT, ventricular tachycardia.

### 3.2 The Role of Left Ventricular Ejection Fraction

Pursuing the purpose of the study and for a more efficient organization we divided our patients into two groups regarding the LVEF after MI and monitored them at 40 days after the MI (one group with patients with LVEF <30% and one group with LVEF between 30–35%) (Table 3). In the first group of patients (LVEF <30% after MI), the mean LVEF after MI was initially 18.93 ± 4.99% (10%–28%), with an increase to a mean of 22.18 ± 4.53% (10%–29%) after a period of 40 days. We conducted a Pearson’s R correlation between the two groups and we obtained a positive and statistically significant correlation (*p *< 0.001 and r – 0.547) resulting in a persistence of LVEF under 30% at 40 days after the MI in the first group of patients. Therefore, all patients remained in the reduced ejection fraction heart failure category and presented an indication of ICD implant 40 days after the acute MI, despite optimal medical therapy.

**Table 3.** **Statistical description of the LVEF in patients evaluated after 40 days since MI**.

Group 1 — Patients with LVEF under 30% after MI							
	N	Min	Max	Mean	St dev	*p*	R
LVEF after MI (before discharge)	55	10	28	18.93	4.999	<0.001	0.547
LVEF at 40 days post-MI	55	10	29	22.18	4.538		
Group 2 — Patients with LVEF between 30% and 35% after MI							
	N	Min	Max	Mean	St dev	*p*	R
LVEF after MI (before discharge)	15	30	34	31.73	1.335	0.02	0.78
LVEF at 40 days post-MI	12	30	34	32.33	1.497		

ICD, implantable cardiac defibrillator; LVEF, left ventricular ejection fraction; MI, myocardial infarction.

In the group of patients with LVEF between 30% and 35% after MI, the mean LVEF after MI was initially 31.73 ± 1.33% (30%–34%), with an increase to a mean of 32.33 ± 1.49% (30%–34%) after a period of 40 days. We conducted a Pearson’s R correlation between the two groups and we obtained a positive and statistically significant correlation (*p* = 0.02 and r – 0.78) resulting in a persistence of LVEF between 30%–35% at 40 days after the MI in the second group of patients. It is worth noting that 3 patients presented an ejection fraction over 35% at 40 days post-MI, and thus, only 12 of the initial 15 patients of this group remained in the reduced ejection fraction heart failure category and presented an indication of ICD implant after 40 days.

### 3.3 Incidence of NSVT and ICD Therapies Depending on LVEF

Most of the ICD therapies — appropriate shock or ATP (14.54%) appeared in patients with LVEF <30%, only 1 patient with the LVEF between 30 and 35% requiring an ICD shock. The patients in the group with LVEF <30% also presented a higher percentage of NSVT at initial ECG monitoring (54% vs. 50%) and NSVT at ICD interrogation (80% vs. 66.7%) when compared to patients with LVEF between 30% and 35%. Statistical significance was not reached in neither of the categories – *p *> 0.05. Descriptive statistics can be tracked in Table 4.

**Table 4.** **Incidence of NSVT in ECG monitoring and ICD interrogation depending on the initial evaluation of the LVEF**.

	Patients with LVEF <30%	*p*	Patients with LVEF between 30% and 35%	*p*
	n = 55		n = 12	
NSVT at ECG monitoring, n (%)	30 (54.5)	0.50	6 (50)	0.36
NSVT at ICD interrogation, n (%)	44 (80)	0.30	8 (66.7)	0.17
Non-VT at ECG monitoring, n (%)	25 (45.5)	0.58	6 (50)	0.37
Non-VT at ICD interrogation, n (%)	5 (9.1)	0.34	1 (8.3)	0.21
Appropriate shock or ATP, n (%)	8 (14.54)	0.40	1 (8.3)	0.24

ATP, anti-tachycardia pacing; ICD, implantable cardiac defibrillator; LVEF, left ventricular ejection fraction; NSVT, Non-sustained ventricular tachycardia; VT, ventricular tachycardia.

### 3.4 Initial NSVT Episodes and The Correlation with Appropriate ICD Therapies 

Appropriate ICD therapy was defined as an ATP or shock for tachyarrhythmia determined to be either VT or VF. Appropriate ICD therapy was observed in 9 patients (13.4%). Most of the ICD therapies — appropriate shock or ATP (11.9%) appeared in patients with NSVT at ECG monitoring, only 1 patient in the group without VT at ECG monitoring requiring an ICD shock. The patients in the group with NSVT at initial continuous ECG monitoring also presented a higher percentage of NSVT at ICD interrogation (77.6% vs. 6%) when compared to patients without VT at the Holter ECG monitoring. Still, statistical significance was not reached – *p *> 0.15 (Table 5).

**Table 5.** **Correlation between arrhythmias detected on ECG Monitoring (in the first week after the myocardial infarction) and the associated adverse event**.

	ECG monitoring	NSVT/NSVF on ICD	Appropriate shock or ATP (%)	Death from any cause	Cardiovascular death	SCD	Sustained VT requiring medical intervention/electrical conversion	*p*
	n = 67	56 (83.6%)	9 (13.4%)	5 (7.5%)	4 (6%)	2 (3%)	1 (1.5%)	
No VT	32 (47.8%)	4 (6%)	1 (1.5%)	1 (1.5%)	-	-	-	0.15
n, (%)								
NSVT	35 (52.2%)	52 (77,6%)	8 (11.9%)	4 (6%)	4 (6%)	2 (3%)	1	
n, (%)								

ATP, anti-tachycardia pacing; ICD, implantable cardiac defibrillator; NSVT, Non-sustained ventricular tachycardia; NSVF, Non-sustained ventricular fibrillation; SCD, sudden cardiac death; VT, ventricular tachycardia.

## 4. Discussions

The present study proves the benefit of early ICD implantation in patients with reduced ejection fraction measured immediately post-MI: all our patients with LVEF <30% after infarction remained in the same category and required the implantation of an ICD at 40 days post-event. Also, 12 out of 15 patients with LVEF between 30% and 35% had indication of ICD implant for primary prevention of SCD. We obtained a positive and statistically significant correlation between reduced LVEF after MI and the necessary of ICD at 40 days. It worth mentioning that we excluded another 7 patients, which needed emergency external shock before the 40th day, and these were implanted with an ICD for the secondary prevention of SCD; thus, 96.1% of patients with reduced ejection fraction post-MI have ended-up by receiving an ICD.

### 4.1 The Optimal Timing — About Early ICD Placement 

#### 4.1.1 ICD Indications According to Current Guidelines

Probably the most widely used clinical marker for SCD is LVEF, as ejection fraction under 35% was correlated in multiple trials with an increased risk of SCD [12, 13, 20, 21]. The pivotal studies for evaluation of ICDs in primary prevention were MADIT [12], followed after a few years by the MADIT-II [13]. MADIT evaluated overall mortality in patients with coronary heart disease, a LVEF of under 35% and abnormal electrophysiological (EP) study. The patients were randomized either to ICD or antiarrhythmic drug (amiodarone in most cases). There was a statistically significant (*p* = 0.009) reduction of relative risk of 54% in patients with ICDs. This was followed by the MADIT II trial, which evaluated the survival in patients with prior MI with a LVEF under 30%, with device therapy versus medical therapy. This was the first trial in which an abnormal EP study was not stated as an inclusion criteria. After a 20 months follow-up, the ICD group showed a 31% risk ratio reduction in the primary endpoint which was the reduction of total mortality. As mentioned above, patients within 3 weeks after MI were excluded in MADIT, and within 4 weeks in MADIT-II. Following the publication of the results from these trials, the current guidelines for primary prevention of SCD in ACS patients are as follows:

- Patients with prior MI, LVEF <35% despite optimal medical therapy, NYHA class II–III, and more than 40 days since last MI or 90 days since most recent revascularization (class I, level A).

- Patients with prior MI, LVEF <30% despite optimal medical therapy, NYHA class I, and more than 40 days since last MI or 90 days since most recent revascularization (class I, level A).

As seen in guidelines, the interval of 40 days after MI remains a gray area even though the incidence of VAs is high, SCD remaining the most severe complication. According to VALIANT [14], a trial that enrolled 14.609 patients with an LVEF ≤40% after AMI and demonstrated that 7% of patients experienced sudden death or cardiac arrest over a two-year follow-up period, the highest rate of SCD was in the first 30 days after MI (1.4% per month), with a gradual decrease down to 0.14% after 2 years. 19% of deaths occurred in the first 30 days after AMI, and the risk was highest in patients with an LVEF ≤30% (2.3% per month). In these circumstances, proper management of VAs after ACS is mandatory and consists mainly in ICD therapy, excepting VT/VF in the early stages of MI (24 to 48 hours) that are considered to be reversible with proper medication and prompt revascularization [22].

#### 4.1.2 Studies in The Field of Early ICD Placement

ACS complicated with VAs and SCD are probably a few of the most important health issues in modern medicine. With the advances in defibrillation devices, the threat of SCD has decreased significantly in the last decades. However, question such as patient selection, adequate time of implantation, type of devices and device programming, quality of life after successive high voltage shock cardioversion should be further investigated in order to give the best medical care for cardiovascular patients.

One of the most debated topics in the regard of ICD after ACS involves the adequate time when the device should be implanted. As far as secondary prevention goes, early cardioverter defibrillator implantation is recommended if a malignant VA occurred >48 h after an acute MI, not due to reversible or correctable causes [9]. However, early SCD primary prevention after ACS is still a challenge, as ICD is not recommended in the first 40 days after an MI [9].

Current contraindications regarding ICD implantation early after an ischemic event are based on several RCTs.

The Defibrillator in Acute Myocardial Infarction trial (DINAMIT) [10] evaluated the benefits of ICD placement from the 6th to the 40th day after a MI, in 674 patients which had a LVEF ≤35% (mean 28%) and markers of autonomic dysfunction (low heart rate variability or increased 24 hours average heart rate). The patients were randomized 1:1, consisting in an ICD group and a standard conventional therapy group. After a follow-up of 30 ± 13 months, the primary outcome (mortality from any cause) was not different in the two groups (hazard ratio (HR) 1.08; 95% CI 0.76–1.55;* p* = 0.66), even though the deaths from arrhythmic cause were significantly reduced in the ICD arm - 12 versus 29 in in the control group (HR 0.42; 95% CI 0.22–0.83; *p* = 0.009). However, it is important to note that only 27% of patients enrolled in the study received primary PCI and that there is no report data regarding the use of aldosterone-blocking agents considering that these have shown to reduce mortality among patients with a recent myocardial infarction and LVEF ≤40%. Although there was no statistical significance in respect of the primary outcome, the results were statistically significant regarding the deaths due to arrhythmia. Therefore, prophylactic ICD therapy does not reduce overall mortality in high-risk patients who have recently had a MI, but it reduces death due to arrhythmia. Given the main purpose of an ICD, the results of this trial must be interpreted strictly in terms of the benefit of lowering the arrhythmic mortality and not influencing the mortality from any cause.

The Immediate Risk Stratification Improves Survival (IRIS) [11] trial enrolled 898 patients 5–31 days after MI, with one or both of the following criteria: EF under 40% and a heart rate over 90 bpm or NSVT of 150 bpm or more, one arm receiving ICD (445 patients) and the other conventional therapy (453 patients). In contrast with DINAMIT, 72% of these patients received primary PCI. Similar to the DINAMIT trial, there was no difference in all-cause mortality rate between the 2 groups (HR 1.04; 95% CI 0.81–1.35; *p* = 0.78), but there was a decrease in arrhythmic death in the ICD group (27 vs. 60; HR 0.55; 95% CI 0.31–1.00;* p* = 0.049). The number of non-SCD was higher in the ICD group than the control group (68 vs. 39; HR 1.92; 95% CI 1.29 to 2.84; *p* = 0.001). The increase number of non-SCD deaths might be a consequence of an imbalance in baseline characteristics between the two groups regarding the development of HF, the different response to treatment and the substrate of acute MI, which vary from person to person. Combining SCD with non-SCD the investigators obtained a HR of 1.04 with respect to all-cause mortality, but regarding the type of death, the ICD significantly reduced the rate of SCD (HR 0.55), the true purpose of implantation. Since VAs are the most common cause of SCD these studies have proven useful in this regard [23, 24].

Futhermore, the BEta-blocker STrategy plus ICD (BEST) [25] trial evaluated 143 patients 5–30 days after a MI, with reduced EF (mean EF 31%), in which an EP study was performed to assess the risk of SCD. The patients were randomized in a 2:3 ratio, to two therapeutic strategies: conventional versus ICD implantation in patients with inducible VT at the EP study. This investigation showed a trend of lower mortality in favor for the ICD group: 14% versus 18% in arrhythmic deaths and 20% versus 29.5% in all-cause mortality, but without statistical significance (*p* = 0.3 and *p* = 0.2). In the BEST + ICD trial, the overall mortality of survivors of an acute MI was rather high ***—*** 16% at 1 year and 24% at 2 years ***—*** even when patients received maximal optimal medical therapy. This indicates that the enrolled population truly constitutes a high-risk subgroup of patients with recent MI, who deserve to be identified and protected by preventive measures.

Resuming the above-mentioned studies, which are cited as factors against early (within the first 40 days) ICD implantation, at a closer analysis, these could be as well used exactly for the opposite, all demonstrating the benefit of ICD in reducing mortality of arrhythmic cause. The fact that they did not show a reduced all-cause mortality should not influence the decision of implanting an ICD and reducing at least the arrhythmic SCD, and thus using the ICD for its main purpose — defibrillation therapy. Comparing the results of these studies, with the results of the MADIT and SCD-HeFT, there is an important discrepancy regarding the benefits of ICD in the first 30–40 days after MI. We can all agree that is rather unnatural that ICDs save lives only starting with the 40th day after acute MI, while in the 20th or 30th day is useless or harmful, especially when it is a well-known fact that the risk of arrhythmic SCD is the greatest in the first 30 days after AMI. It is quite a paradox, but one that we all accept; according to it, we guide our medical decisions and the life of our patients and potentially expose them to death which could possibly be avoided.

Even though one may argue that these differences could also come as a result of the fact that the major mechanism of death early after a MI is pump disfunction [13], taking into consideration the high incidence of VAs in the first month after MI and the overall accepted utility of ICD in decreasing the arrhythmic mortality, ICD should prove its benefit in these circumstances. Besides VAs, there are also other cardiac and non-cardiac causes of SCD (ventricular rupture, pericarditis, pulmonary embolus, dissecting aortic aneurysm and intracerebral hemorrhage) in the early MI period, which could not be prevented by ICD, but the proportion in which they contribute to overall mortality post-MI is rather minor.

### 4.2 The Importance of Proper ICD Programming

Another explanation on the increased overall mortality in ICD arms could be the number of inappropriate shocks received, usually for supraventricular tachyarrhythmia, which can accelerate the progression of heart failure [26]. An analysis of the MADIT II population revealed that atrial fibrillation and supraventricular tachycardias accounted for more than 80% of cases of inappropriate shocks, and these shocks doubled the risk of all-cause mortality [27]. This effect of ICD high voltage cardioversion is a result of myocardial depression, proarrhythmic effect and the thromboembolic complications that can appear after shock [28]. Consequently, programming of the devices in order to reduce the impact of the electric shocks on the myocardium and to better discriminate supraventricular arrhythmias would be the path to follow, not contraindicating the ICD implant, which is vital to post-MI patients.

Recent studies demonstrated that many episodes of VT will terminate spontaneously or can be effectively terminated by anti-tachycardic pacing [29, 30]. The MADIT-Reduce Inappropriate Therapy trial enrolled 1500 patients with ICD, which were randomized into 3 groups: one with high rate therapy (a 2.5-second delay before the initiation of therapy at a heart rate of ≥200 beats per minute), one with delayed therapy (a 60-second delay at 170 to 199 beats per minute, a 12-second delay at 200 to 249 beats per minute, and a 2.5-second delay at ≥250 beats per minute) and one group with conventional therapy (with a 2.5-second delay at 170 to 199 beats per minute and a 1.0-second delay at ≥200 beats per minute). The study showed that the high rate therapy and the delayed therapy were both associated with reduction in inappropriate therapy and all-cause mortality [15]. Therefore, current guidelines recommend for primary prevention patients to increase the rate cutoff up to 200 bpm, using more than 1 detection zone and programming ATP before and during charging [31]. Another important factor in avoiding inappropriate shocks is adequate discrimination of supraventricular tachycardias. In this aspect, device manufacturers have come with multiple discrimination algorithms that can provide a rhythm classification of ventricular or supraventricular. These are based on morphological discriminators, stability and sudden onset algorithms. Furthermore, dual-chamber devices can directly compare atrial vs. ventricular rates. In this sense, the Optimal Anti-Tachycardia Therapy in Implantable Cardioverter-Defibrillator Patients Without Pacing Indications (OPTION) trial which randomized single-chamber vs. dual-chamber ICD patients, revealed that 4.3% of patients in the dual-chamber setting group compared with 10.3% in the single-chamber setting group experienced inappropriate shocks (*p* = 0.015) [32]. In order to improve the detection capacities of single chamber devices and to avoid the risks and costs of adding another lead, defibrillation leads with floating atrial electrodes have been designed, with promising preliminary results [33].

The ICD doesn’t prevent death from progressive pump failure and may just allow for change in the mode, but also in the timing of death. Whereas internal shocks, appropriate or inappropriate, are associated with increased rate of death, ATP-treated arrhythmias are not increasing mortality. Modern programming, with more aggressive ATP, along with extended detection and the use of discrimination algorithms can help reduce the frequency of inappropriate shocks, and save lives [34]. Although SCD-HeFT demonstrated the superiority of ICD therapy, by reducing the mortality by 23% when compared to amiodarone, there are multiple studies showing that the use of antiarrhythmics, especially the association between amiodarone and a beta-blocking agent dramatically reduced shocks [35, 36, 37].

An additional ICD related factor incriminated for the high rates of overall mortality in prevention trials, is the right ventricle stimulation by the ICD. It induces non-physiological depolarization which has been proven to have a negative hemodynamic effect and can be an additional factor responsible for heart failure deterioration [38]. As a result, no rate-responsive pacing modes should be used, and current guidelines even recommend the implantation of a CRT-D device in patients requiring both SCD prevention and long-term anti-bradycardia pacing (more than 40% right ventricular pacing) [9, 39].

However, implanting a device 40 day earlier or later, can’t make the difference in the number of heart failure caused by significant RV pacing or inappropriate shocks; a supplementary pacing time of 40 days could not lead to increased all-cause mortality due to pump failure in early ICD implantation trials. So, still, despite the negative effect of pacing and shocks, we could not find the factor that conducts to this huge difference in mortality when the ICD is implanted before, respectively after the 40 days since MI, a plausible answer being that it could be all a matter of interpreting statistics. Other explanations for the negative results in early ICD trials were advanced: general anesthesia and defibrillation testing [DFT] early after MI may led to unknown effects on cardiac remodeling. ICDs were systematically tested at implant both in IRIS and DINAMIT [40]. However, a trial that randomized patients to DFT versus no DFT, showed that DFT did not increase all-cause mortality during the mean follow-up of 3.1 years [41].

A recent trial that reflects the advances in ICD technologies and programming, and also focuses on early device implantation after revascularization in the setting of ACS was the DAPA (Defibrillator After Primary Angioplasty) [42] trial. This prospective randomized multicentric study enrolled 262 patients who underwent primary PCI between 30 and 60 days after ST-elevation myocardial infarction (median 50 days), which also had at least one risk factor such as: VF, EF lower than 30%, Killip class 2 or higher or TIMI flow less than 3 after primary PCI. The patients were assigned to the standard care or the ICD arm within 7 days after randomization. All ICDs had the following programming: high voltage electrical shock cardioversion for VT or VF at a cut-off heart rate of ≥190 beats per minute (ATP burst during charging). The monitor zone was programmed to document VT with ventricular rates between 160 and 190 beats per minute. The ICD group presented significantly decreased rate of all-cause mortality of 5% vs. 13% after 3 years follow-up and 18% vs. 38% at the 9-year follow-up. The cardiac mortality was also significantly lower in the ICD group, while there were no differences regarding non-cardiac deaths between the 2 groups. This study shows earlier implantation of and ICD is safe if proper patient selection together with the advances in device programming and technologies are combined. However, in order to change the current guidelines these results should be confirmed by future larger trials, which should also evaluate the percentage of arrhythmic deaths prevented in the first days/weeks after a MI.

### 4.3 The Wearable Cardioverter-Defibrillator 

Albeit there are multiple proves that early ICD implantation may be the appropriate solution, in order to respect the currently in use guidelines, there is a clear need for a non-ICD strategy to protect patients against the occurrence of SCD. The wearable cardioverter-defibrillator (WCD), approved by the FDA and recommended with a IIb class of indication in the ESC and ACC/AHA/HRS guidelines, appears to be the solution for patients with HF who are at risk of SCD for a limited period or as a bridge to an implantable device. The WCD is not by far a perfect alternative to an ICD. It has the ability to deliver high-voltage defibrillation electric shocks and doesn’t require external intervention from a bystander, however it lacks functions such as anti-tachycardia pacing and post-shock anti bradycardia pacing. The experience with the WCD has been described in numerous studies published from 2001 to 2018, including one of the largest studies on 8.453 patients from the Zoll Registry: 133 patients (1.6%) received 309 appropriate shocks with a rate of 91% successful shocks; 75% of WCD therapy occurred in the first month after AMI [43]. The Vest Prevention of Early Sudden Death Trial (VEST), focused only on primary prevention of SCD using an WCD in patients with reduced EF, 30 to 40 days after a MI, when an ICD is not yet indicated. A total of 2348 patients were included, randomized 2:1 and the primary outcome was arrhythmic death. The results of the study showed no difference between the device group and control group (1.6% vs. 2.4% arrhythmic deaths, *p* = 0.18), but there was a significant difference regarding overall mortality between the two groups (3.1% in the device group vs. 2.4% in the control group, *p* = 0.04). These contradictory results were based on design limitations of the study, where the primary outcome was changed from all-cause mortality to arrhythmic mortality after patient enrollment, and furthermore the cause of death was established by an independent panel who was unaware of this change and did not receive data from the WCD. This could have led to an improper classification of the cause of death and thus to the contradictory results of the study. On the other hand, the study raised one of the main concerns regarding WCD, which is patient compliance. In the VEST study the median daily wear time of a WCD was 14 h, and investigators even reported that 3 out of 4 patients who died in the WCD group did not wear the device at the moment of death [44]. The low number of studies, with contradictory results have made the acquisition of devices such as wearable ICDs controversial for healthcare systems, especially in developing countries where also transvenous ICD implantation is underfunded. In these situations, physicians are required to evaluate and monitor carefully patients who could be candidates for early ICD implantation. The largest available meta-analysis including 33.242 patients, revealed that appropriate WCD treatments, appropriate and inappropriate WCD shocks occurred at a rate of 7/100-persons, 5/100-persons, and respectively 2/100-persons over a three-month time period. Interestingly, patients with ischemic cardiomyopathy in VEST had a higher incidence of appropriate shock (11/100-patients) when compared with patients with ischemic cardiomyopathy in the non-VEST (1/100-patients). A difference that only underlines the non-uniformity of WCD trials. Notwithstanding the evidence, to this day, WCD continues to be prescribed, and, in certain institutions, has become the standard of care. As a conclusion regarding the prescription of WCD, Masri *et al*. [45] made an observation that encompasses the use of WCD: “This practice pattern is likely driven by the finality of SCD and partly by fear of litigation, despite the absence of data to support it”.

### 4.4 Recovery of Left Ventricular Ejection Fraction After Myocardial Infarction

The current indication for ICD placement 40 days after the acute MI is conditioned by a LVEF of under 35%. The result of the current study shows that all patients with EF of under 30% at the time of MI remained in the category of reduced ejection fraction 40 days later and benefit of ICD implant.

In regard to the recovery of LVEF after acute myocardial infarction treated by PCI, Ottervanger [46] followed 600 consecutive patients with AMI treated with primary angioplasty. LVEF was measured at day 4 and 6 months after PCI. The mean EF at discharge was 43.7% (at 4 days after AMI), whereas the mean EF after 6 months was 46. 3%. During the 6 months, the mean relative improvement in LVEF was 6%. The authors comment that within the day 4 after angioplasty, the stunning in smaller infarction was partially resolved, while in larger infarction the stunning period was prolonged beyond this period. Reibis *et al*. [47] included 277 consecutive patients with LVEF ≤40% at approximately 1 month after AMI. The increase in LVEF in the study population was also moderate at 6%. It worth mentioning that the authors affirm that the raw LVEF measurements suggest more marked changes in the preselected individuals, but the change in mean values in subgroup populations are influenced by regression towards the mean (RTTM), which reflects a statistical effect describing the relationship between two linked measurements; after taking RTTM into consideration, the improvement of LVEF was even less, and, most importantly, it was not significant for clinical decisions, even though major proportion of patients had a mild increase of LVEF as measured after revascularization. The improvement of LVEF is rather a result of interindividual variability, intraindividual variability and RTTM, with the slight addition of the actual increase of LV function. Nevertheless, clinicians tend to interpret the observed changes as real improvements and thus they may tend to draw excessively optimistic conclusions in regard to the clinical course of the patients, while the real (as opposed to the apparent) recovery of LVEF after early post-MI revascularization proved to be sooner mild. Even after complete revascularization, in heart failure patients, the systolic dysfunction persists, remaining largely unchanged. According to the expectations, it is rather improbable that the true LV function will return to normal in patients with a damaged post-AMI ventricle.

Furthermore, even if complete revascularization is essential for improving left ventricular function, the recovery of impaired ejection fraction in post-myocardial patients is usually modest and this can be expected only in stunned or hibernating myocardial segments [48]. Usually, stunned myocardium is likely to show an early improvement of function, the recovery of the myocardium from stunning occurring within 2 weeks in patients treated with reperfusion therapy [49, 50]. Hibernating myocardium may take a longer time to completely recover, with further improvement that could take until 14 months, as shown in some studies [51]. Hibernation appears both in the infarcted areas, as also in areas remote from the area of infarct, but still adjacent to it. Late post-reperfusion improvement in myocardial contractility, could be evidenced in these areas by several parameters [52].

These observations in a broad myocardial infarction population provide relevant data when considering the appropriate strategies and therapies for prevention of SCD. Returning to the initial question, how long should we wait until ICD placement, it is clear we can’t afford to wait until the full recovery of the hibernating myocardium. Since patients with stunned myocardium have shown myocardial recovery in approximately 2 weeks, do we really have an argument for the 40-days delay? It seems we rather have arguments against this prolonged waiting time.

### 4.5 NSVT as Predictor of Cardiovascular Adverse Events

Although NSVT was statistically associate to subsequent adverse events in multiple studies, in our population, we could not reach statistical significance. Still, most of the ICD therapies (internal shock or ATP) appeared in patients with NSVT at ECG monitoring. The patients in the group with NSVT at continuous ECG monitoring also presented a higher percentage of NSVT at ICD interrogation (77.6% vs. 6%) when compared to patients without VT at the initial ECG monitoring.

The Metabolic Efficiency with Ranolazine for Less Ischemia in Non–ST-Elevation Acute Coronary Syndrome–Thrombolysis in Myocardial Infarction 36 (MERLIN-TIMI 36) [53] study included 6560 patients with a non–ST-elevation ACS, of which 6345 patients (97%) had continuous ECG recordings for the first 7 days after randomization. SCD (n = 121) was assessed over a median follow-up of 1 year. The risk of SCD was significantly greater in patients with NSVT lasting 4 to 7 beats (SCD, 2.9%; adjusted hazard ratio, 2.3; *p *< 0.001) and in patients with NSVT lasting at least 8 beats (SCD, 4.3%; adjusted hazard ratio, 2.8; *p* = 0.001). This effect was independent of the patient’s baseline characteristics or LVEF. In the Platelet Inhibition and Patient Outcomes (PLATO) trial, ECG monitoring was also carried out during the first 7 days after myocardial infarction in 2866 patients and repeated at day 30 in 1991 patients. NSVT detected in both the acute and convalescent phases and was significantly associated with an increased risk of cardiovascular death. Similar to VALIANT trial, the greatest risk of cardiovascular death was in the first 30 days, while in the convalescent phase, the risk of death associated with NSVT remained elevated for approximately 5 months [54].

The literature offers conflicting conclusions on the relationship between NSVT and adverse cardiovascular events, some previous studies of patients with STEMI demonstrating an independent relationship between VT and cardiovascular events [55, 56, 57] whilst others did not link VT to subsequent adverse events [58, 59]. Al-Khatib *et al*. [60], in his analysis of over 26,000 patients with Non-STEMI, proved that sustained VAs were independently associated with increased 30-day and 6-month mortality, but the relationship between NSVT and outcomes was not examined. Mäkikallio *et al*. [61], followed 2130 patients with STEMI and non-STEMI by continuous ECG recordings that lasted for 24 hours and were performed in the first 4 weeks after the ACS, and demonstrated that NSVT was associated with SCD.

### 4.6 Reduced Ejection Fraction — Still The Main Predictor of SCD

In the long run, reduced LVEF remains the single best predictor of SCD. A systematic review of 12 randomized trials and 76 observational studies, which included more than 100,000 patients, showed that ICDs are effective in adults with heart failure with reduced EF and that the benefits extend beyond trial populations [62]. All the data and results presented above lead to the necessity of revising the guidelines contraindication of implanting an ICD early post-MI. Moreover, we wonder, should the current guidelines recommendations be based on the results of individual separate studies, rather than on the results of the already available meta-analyzes? Are we recommending ICDs to reduce arrhythmic mortality, or are we seeing them as a universal remedy and we will continue seeking the capability of ICDs in reducing the all-cause mortality, including the non-cardiovascular one, instead of using the ICD for its main purpose — defibrillation?

## 5. Limitations

Our study was a retrospective, nonrandomized, single-center research, following a relatively restrain number of patients. Another limitation of the study was the medium follow-up period of 38 months, with the follow-up of the last included patients of only 7 months, while some patients had a follow-up of up to 57 months. Within this period of time, new heart failure medications have become available. This may have caused heterogenicity in the study population, but this limitation did not majorly influence the aim of the study, since the purpose was the need of ICD 40 days after the MI. Larger trials and more representative multi-center registry data are needed to confirm our findings.

## 6. Conclusions

The adequate time of ICD implantation for the primary prevention of SCD is disputable. The present study shows the correlation between reduced ejection fraction post-MI and the need for ICD implant 40 days later. Considering also the already proved risk of increased arrhythmic SCD in the first month after MI and reflecting dipper on the results of studies on early ICD implantation, our paper brings supplementary arguments to question the 40 days waiting time post-MI and demonstrate the futility and risks that this delay brings.
